# Cladribine Alters Immune Cell Surface Molecules for Adhesion and Costimulation: Further Insights to the Mode of Action in Multiple Sclerosis

**DOI:** 10.3390/cells10113116

**Published:** 2021-11-10

**Authors:** Tobias Moser, Lena Hoepner, Kerstin Schwenker, Michael Seiberl, Julia Feige, Katja Akgün, Elisabeth Haschke-Becher, Tjalf Ziemssen, Johann Sellner

**Affiliations:** 1 Department of Neurology, Christian Doppler Medical Center, Paracelsus Medical University, 5020 Salzburg, Austria; t.moser@salk.at (T.M.); k.schwenker@salk.at (K.S.); m.seiberl@salk.at (M.S.); j.feige@salk.at (J.F.); 2 Department of Neurology, Multiple Sclerosis Center, Center of Clinical Neuroscience, Carl Gustav Carus University Hospital, Technical University Dresden, 01307 Dresden, Germany; Lena.Hoepner@uniklinikum-dresden.de (L.H.); Katja.Akguen@uniklinikum-dresden.de (K.A.); tjalf.ziemssen@uniklinikum-dresden.de (T.Z.); 3 Department of Laboratory Medicine, Paracelsus Medical University, 5020 Salzburg, Austria; e.haschke-becher@salk.at; 4 Department of Neurology, Klinikum rechts der Isar, Technische Universität München, 80333 München, Germany; 5 Department of Neurology, Landesklinikum Mistelbach-Gänserndorf, 2130 Mistelbach, Austria

**Keywords:** cladribine, multiple sclerosis, adhesion molecules, costimulatory molecules, immune cells, LFA-1, ICAM-1, CD154

## Abstract

Cladribine (CLAD) is a deoxyadenosine analogue prodrug which is given in multiple sclerosis (MS) as two short oral treatment courses 12 months apart. Reconstitution of adaptive immune function following selective immune cell depletion is the presumed mode of action. In this exploratory study, we investigated the impact of CLAD tablets on immune cell surface molecules for adhesion (CAMs) and costimulation (CoSs) in people with MS (pwMS). We studied 18 pwMS who started treatment with CLAD and 10 healthy controls (HCs). Peripheral blood mononuclear cells were collected at baseline and every 3 months throughout a 24-month period. We analysed ICAM-1, LFA-1, CD28, HLADR, CD154, CD44, VLA-4 (CD49d/CD29), PSGL-1 and PD-1 with regard to their expression on B and T cells (T helper (Th) and cytotoxic T cells (cT)) and surface density (mean fluorescence intensity, MFI) by flow cytometry. The targeted analysis of CAM and CoS on the surface of immune cells in pwMS revealed a higher percentage of ICAM-1 (B cells, Th, cT), LFA-1 (B cells, cT), HLADR (B cells, cT), CD28 (cT) and CD154 (Th). In pwMS, we found lower frequencies of Th and cT cells expressing PSGL-1 and B cells for the inhibitory signal PD-1, whereas the surface expression of LFA-1 on cT and of HLADR on B cells was denser. Twenty-four months after the first CLAD cycle, the frequencies of B cells expressing CD44, CD29 and CD49d were lower compared with the baseline, together with decreased densities of ICAM-1, CD44 and HLADR. The rate of CD154 expressing Th cells dropped at 12 months. For cT, no changes were seen for frequency or density. Immune reconstitution by oral CLAD was associated with modification of the pro-migratory and -inflammatory surface patterns of CAMs and CoSs in immune cell subsets. This observation pertains primarily to B cells, which are key cells underlying MS pathogenesis.

## 1. Introduction

Multiple sclerosis (MS) is characterized by the peripheral formation of autoreactive lymphocytes with encephalitogenic potential [1]. The migration of pathogenic cells from the periphery to the central nervous system (CNS) is restricted by the blood–brain barrier (BBB), which physiologically preserves local homeostasis and an optimal environment for neuronal function. The interaction between cell-bound adhesion molecules (CAMs) expressed by activated leukocytes and their cognate ligands present on the endothelial cells of the BBB plays a central role in the transmigration of immune cells to and within the CNS [2,3]. CAMs comprise members of the integrin (leucocyte function associated molecule-1 (LFA-1), very-late antigen-4 (VLA-4, consisting of CD49d and CD29), immunoglobulin (intercellular adhesion molecule-1 (ICAM-1)) and sialomucin (P-selectin glycoprotein ligand-1 (PSGL-1)) superfamily [2,4]. Besides promoting transmigration, CAMs are indispensable for sustained cell–cell contact within the immunological synapse (IS) [4]. Under physiological conditions, the IS optimizes pathogen control, while dysregulation is central to the proliferation of autoreactive T helper cells and the subsequent autoimmune processes [5,6]. The IS is the interface between an antigen-presenting cell or a target cell, and an immune cell (i.e., a T, B or natural killer cell) [7]. Autoreactive T cell clones arise from direct T helper (Th)–B cell communication via T cell receptor (TCR)–human leukocyte antigen (HLA) engagement. The role of HLA is underscored by the identification of HLA-DRB1*1501 as the major genetic risk factor for MS [8]. Elicitation of memory lymphocytes requires the pairing of costimulatory molecules within the IS [4]. Costimulatory signals (CoSs) involved in the pathogenesis of MS are CD80/CD86 on antigen-presenting cells (APCs) and their cognate ligand CD28 (on naïve lymphocytes), as well as the binding of CD154 (on Th cells) to CD40 (on APCs) [9,10].

There are several lines of evidence for the involvement of adhesion molecules in the immunopathogenesis of and as drivers of inflammation in MS [11,12,13,14,15,16]. The role of CAMs in MS is further highlighted by the mode of action of natalizumab, a monoclonal antibody targeting VLA-4, which efficiently hampers CNS recruitment of immune cells and inflammatory disease activity [17]. The importance of the CoSs and CAMs in the pathogenesis of CNS autoimmunity is also supported by the findings from animal models, as the induction of experimental autoimmune encephalomyelitis (EAE) is suppressed by antibodies targeting ICAM-1, CD154 and VLA-4, as well as pathways involving CD28 and CD44 [18,19,20,21,22,23,24,25].

Even though MS is a chronic autoimmune disorder of the CNS, peripheral blood (PB) represents an accessible biological sample and provides a “window” into the immune signatures associated with the disease. In fact, a pro-migratory surface expression profile of CAMs was found in the peripheral blood mononuclear cells of pwMS, which was attenuated by treatment with glatiramer acetate, a parenteral disease-modifying therapy (DMT) [26]. Dimethyl fumarate (DMF), an oral DMT, reduces the costimulatory potential via CD40 inhibition on APCs [27,28].

Cladribine (CLAD, Mavenclad) is a deoxyadenosine analogue pro-drug which preferentially depletes lymphocytes and is approved for the treatment of active MS. CLAD treatment in MS requires two treatment-week cycles per year. One cycle is given at the beginning of the first month and another cycle at the beginning of the second month of Years 1 and 2. This treatment has the potential for no further treatment in Years 3 and 4, but the exact mechanism of this persistent efficacy remains to be elucidated [29]. One hypothesis is that the adaptive immune system may reconstitute with a lower autoimmune profile following semi-selective depletion by CLAD. Immunological consequences seen after CLAD treatment in the peripheral blood include a marked and long-term depletion of B cell subsets [30,31,32]. A less extensive but likewise long-term depletion of CD4+ T cell subsets and non-classical Th17 cells is another feature of CLAD treatment [30,33]. In vitro, CLAD decreases the migratory capacity of T cells and, to a lesser extent, of monocytes [34]. In pwMS, CLAD intake was associated with a reduction of soluble ICAM and E-selectin concentrations in the peripheral blood [35]. Thus, CLAD might achieve, at least in part, its clinical and paraclinical efficacy by modulating the adaptive immune responses on the basis of CAMs and CoSs on immune cell subsets.

This exploratory study aimed to corroborate the peripheral blood immune cell signature in pwMS on the basis of cell-bound adhesion and stimulatory molecules. Moreover, we studied the impact of oral CLAD on this pattern over the course of 24 months.

## 2. Materials and Methods

### 2.1. Study Cohorts

We recruited 18 pwMS who were started on CLAD treatment in two European MS centres (Christian Doppler Medical Center Salzburg, Austria and Carl Gustav Carus University Hospital, Technical University Dresden, Germany) and 10 age-matched healthy controls (HCs). Blood was drawn twice from each HC at intervals of several months. Acute infections were excluded prior to sampling.

We assessed the expression of CAMs and CoSs on CD4+, CD8+ and CD19+ lymphocytes from HCs and compared them with the baseline (BL) values of pwMS. To investigate CLAD-associated changes, peripheral blood mononuclear cells (PBMC) were collected every 12 weeks (±4 weeks) for 24 months from pwMS. Demographic data and the medical history, as well as relapses and changes in the Expanded Disability Status Score (EDSS) within the observational period, were obtained (Table 1 and Table A1). The use and dosage of CLAD was according to the guidance provided by the European Medical Agency [36]. Treatment with CLAD was only started when lymphocyte counts were in the normal range. Study inclusion required a minimum of 4 weeks of steroid treatment. All patients provided signed informed consent before enrolment. The study was conducted according to the Declaration of Helsinki and was evaluated by the local ethics committees.

### 2.2. Antibodies and Flow Cytometry

PBMCs were isolated and processed according to standard operating procedures (SOPs), as described before [37]. All samples were analysed in a single batch in order to maximize precision and avoid data acquisition bias. In short, PBMCs were isolated from heparinized or citrated blood and cryo-preserved at −130 °C. After thawing, cells were incubated with the viability marker Zombie Green—Alexa488 (Biolegend, San Diego, CA, USA) and washed with a FACS buffer (phosphate buffered saline, 0.2% foetal calf serum and 0.02% sodium azide). The resulting cells were stained with fluorescence-labelled antibodies (BD Biosciences, Franklin Lakes, NJ, USA): CD3-APC-H7, CD4-PE-C7, CD8PerCPCy5, CD19-BV510, ICAM-1-APC, PD-1-BV605, CD28-PE-Cy5 and CD29-BV786; Biolegend, San Diego, CA, USA: CD44-BV421, LFA-1-A700, HLADR-PE, PSGL-1-PE-CF594 and CD49d-BV711). For intracellular staining (BD Biosciences: CD3-APC-H7, CD4-BV510; Biolegend: CD154-PerCPCy5), cells were stimulated with 10 ng/mL phorbol myristate acetate (PMA, Sigma-Aldrich, St. Louis, MO, USA) and 1 µg/mL ionomycin (Sigma-Aldrich) and supplemented with 0.2 µM monensin (Biomol, Hamburg, Germany) for 6 h at 37 °C, fixed with paraformaldehyde (PFA) and permeabilized with saponin. All samples were rinsed with the FACS buffer and measured on a LSR-Fortessa instrument (BD Biosciences). Evaluation of the FACS data was performed by FACS-Diva Software (BD Biosciences). Immune cell populations were gated as Th (CD3+CD4+), cytotoxic T (cT, CD3+CD8+) and B cells (CD3-CD19+). To evaluate the kinetics of CAMs and CoSs associated with CLAD intake over time, we used two approaches. In addition to changes in the proportions (frequencies of Th, cT and B cells among the respective parent populations expressing the surface markers), we evaluated alterations in the signal intensity (in terms of the mean fluorescence intensity, MFI [38]) of each cell-bound marker on the surface of the entire respective lymphocyte subset. The gating strategy was population-based (Figure A2) and carried out by two independent investigators. No FMOs were performed. Both evaluation methods were also used to detect differences in surface protein expression between the two groups. Absolute numbers were available for CD154+ Th cells; for this evaluation, only significant differences at 24 months from CLAD initiation are shown.

### 2.3. Statistical Analysis

Statistical analysis was performed by IBM SPSS Statistics 25 Software for Windows (Version 25.0; IBM Corporation, Armonk, NY, USA). The Mann–Whitney U-test for independent samples was used to detect differences between the baseline values of pwMS and healthy blood donors. To evaluate changes in CAMs and CoSs over time, we used generalized linear mixed models (GLMM) with Bonferroni’s correction. GLMM was also used to test for intra-individual significant differences within HCs to determine stability of CAM and CoS expression. Data were tested for a normal distribution with the Shapiro–Wilk test. If the data exhibited a right-skewed distribution pattern, a gamma distribution was used. Significant differences for the CLAD-associated effects on the expression pattern of CAMs and CoSs on lymphocytes were calculated every 3 months throughout the 2-year observational period and compared with pre-treatment values (BL). The evaluation of CD154 expression was made after cell stimulation in a separate experiment, which allowed the possibility to calculate absolute cell numbers. In this regard, CD4+ cell counts were extracted from complete blood cells. *p*-values were considered significant as follows: * *p* ≤ 0.05, ** *p* ≤ 0.01, *** *p* ≤ 0.001 and **** *p* ≤ 0.0001. Graphs were created with GraphPad PRISM8 (GraphPad Software, San Diego, CA, USA).

## 3. Results

Three patients (17%) showed disease activity (clinical ± MRI) during the study period and all three were treated with intravenous steroids for 5 days (Table A1). No serious adverse events related to CLAD were reported. Two patients developed transient Grade 3 lymphopenia at 3 months from the start of the second cycle (month 15) but no Grade 4 lymphopenia was observed.

Over time, we had relevant dropouts. We lost 2two pwMS in Month 12 (#5 and #6), six in Month 15 (#11, #12, #13, #14, #15 and #16), one in Month 18 (#10) and five in Months 21/24 (#4, #7, #8, #9 and #18). Only one pwMS (#6) left the study because of disease activity and switched to another DMT (in Month 12). In the other patients, either CLAD intake in the second year was postponed or the follow-up visit was cancelled due to precautions related to the COVID-19 pandemic. Thus, the pre-scheduled sampling was restricted to 16 patients in Month 12, in 10 patients in Month 15, in nine patients in Month 18 and in four in Months 21/24.

### 3.1. MS vs. HCs

For each healthy participant, blood was taken twice (on average, 5.8 months apart). We found no relevant interindividual alterations in the expression of CAMs and CoSs on immune cells by longitudinal sampling, indicating that the surface pattern in terms of frequency and MFI was stable in the healthy population. The proportions of the main lymphocyte subsets did not differ between the two cohorts (Table 1).

**Figure 1:**
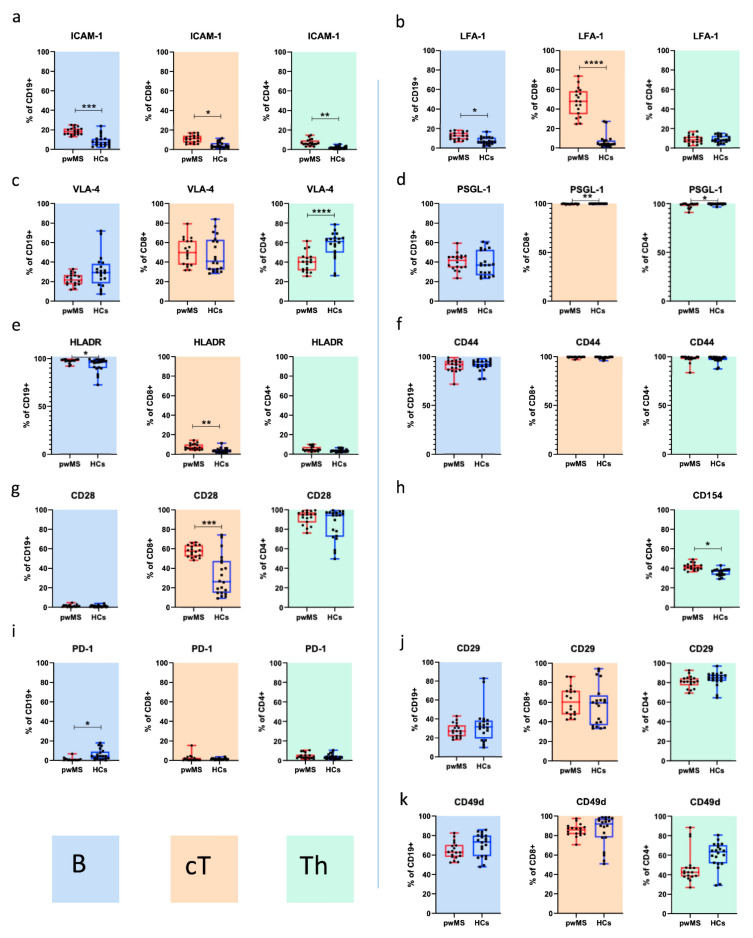
Baseline characteristics for adhesion and costimulatory surface molecules in healthy controls (HCs) and patients with MS (pwMS) (**a**–**k**) as measured by flow cytometry: frequencies of B cells/CD19+ (blue graphs), cytotoxic T cells/CD8+ (brown graphs) and T helper cells/CD4+ (green graphs). For intracellular staining with CD154, cells were stimulated for 6 h, fixed and permeabilized. We found a pro-migratory and -inflammatory profile, characterized by a higher percentage of ICAM-1 (CD19+, CD4+ and CD8+ (**a**)), LFA-1 (CD19+ and CD8+ (**b**)), HLADR (CD19+ and CD8+ (**e**)), CD28 (CD8+ (**g**)) and CD154 (CD4+ (**h**)) expressing immune cells. Legends: Box-and-whisker plots show the median, interquartile range and minimum–maximum range. * *p* ≤ 0.05, ** *p* ≤ 0.01, *** *p* ≤ 0.001 and **** *p* ≤ 0.0001.

**Figure 2:**
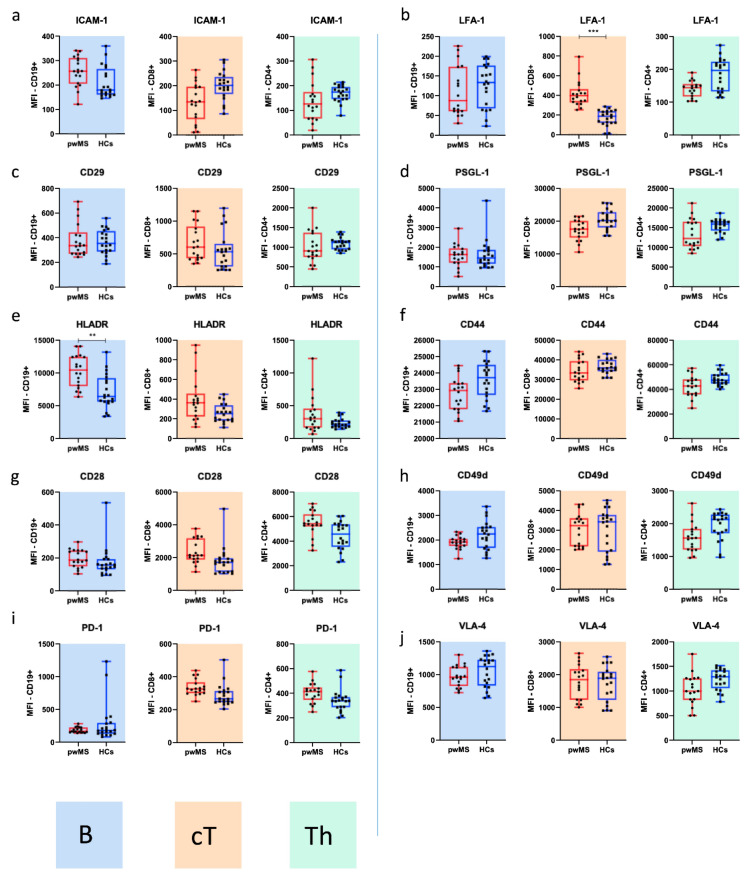
Baseline characteristics for adhesion and costimulatory surface molecules in healthy controls (HCs) and patients with MS (pwMS) (**a**–**j**): surface density (MFI) of B cells/CD19+ (blue graphs), cytotoxic T cells/CD8+ (brown graphs) and T helper cells/CD4+ (green graphs). The molecular surface expression patterns were analysed by flow cytometry. Surface intensity staining for LFA-1 (CD8+ (**b**)) and HLADR (CD19+ (**e**)) was increased within the MS cohort. Legend: Box-and-whisker plots show the median, interquartile range and minimum–maximum range. ** *p* ≤ 0.01 and *** *p* ≤ 0.001.

**Figure A1:**
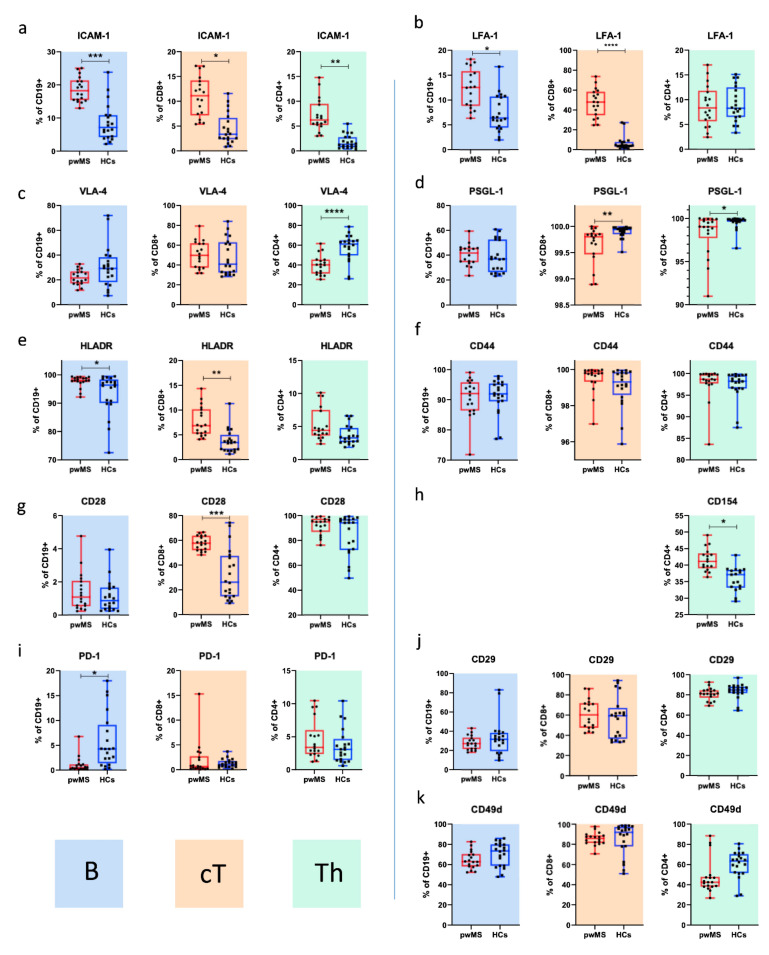
Baseline characteristics of adhesion and costimulatory surface molecules in HCs and pwMS (**a**–**k**) as measured by flow cytometry: frequencies of B cells/CD19+ (blue graphs), cytotoxic T cells/CD8+ (brown graphs) and T helper cells/CD4+ (green graphs). For intracellular staining with CD154, cells were stimulated for 6 h, fixed and permeabilized. We found a pro-migratory and -inflammatory profile characterized by a higher percentage of ICAM-1 (CD19+, CD4+ and CD8+ (**a**)), LFA-1 (CD19+ and CD8+ (**b**)), HLADR (CD19+ and CD8+ (**e**)), CD28 (CD8+ (**g**)) and CD154 (CD4+ (**h**)) expressing immune cells. Legends: Box-and-whisker plots show the median, interquartile range and minimum–maximum range. * *p* ≤ 0.05, ** *p* ≤ 0.01, *** *p* ≤ 0.001 and **** *p* ≤ 0.0001.

**Figure A2:**
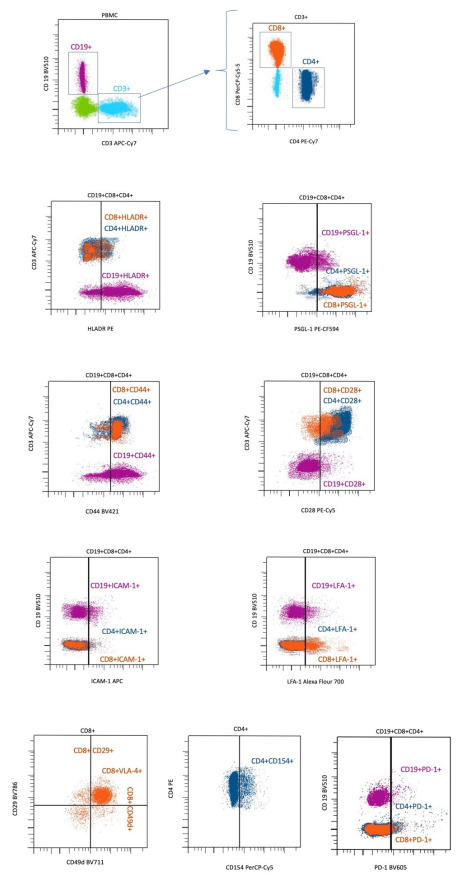
Gating strategy.

We then investigated the differences in the surface expression pattern of molecules involved in cell adhesion and costimulation on T helper cells (CD4+, Th), cytotoxic T cells (CD8+, cT) and B cells (CD19+) between the two cohorts. For this purpose, we compared samples taken from pwMS at BL (*n* = 18) with those of age-matched HCs (*n* = 10) and evaluated differences in the frequencies (Figure 1 and Figure A1**)** and MFI (Figure 2).

#### 3.1.1. Adhesion Molecules

For adhesion molecules, we found that the frequencies of ICAM-1+ lymphocytes were significantly increased in pwMS, and the differences were most pronounced among B cells (CD8+: 11% vs. 4.4%, *p* = 0.028202, CD4+: 7.1% vs. 1.9%, *p* = 0.003179; CD19+: 18.2% vs. 8.8%, *p* = 0.000439; Figure 1a and Figure A1a). The frequencies of LFA-1+ CD19+ and CD8+ lymphocytes were also significantly increased in pwMS (*p* = 0.020467 and *p* = 0.000000022217, respectively; Figure 1b and Figure A1b). We next investigated the expression patterns of CD49d, CD29 and VLA-4, which were gated as CD49d/CD29 double-positive cells. While we found differences for single CD49d and CD29 positive cells (Figure 1j,k and Figure A1j,k), the frequencies of Th cells expressing VLA-4 were significantly lower in pwMS compared with HCs (38.6% vs. 57.6%, *p* = 0.00082; Figure 1c and Figure A1c). High proportions of cT and Th cells across both cohorts were found to express PSGL-1, and PSGL-1 frequencies were higher in HCs (Th 99.9% vs. 99.6%; *p* = 0.004 and cT 99.5% vs. 98.2%; *p* = 0.047; Figure 1d and Figure A1d). Regarding MFI **(**Figure 2), we found an increased surface density of LFA-1 on cT cells from pwMS (*p* = 0.000065, Figure 2b).

#### 3.1.2. Costimulatory Molecules

We next examined the expression profile of stimulatory and inhibitory molecules, including HLADR, CD28, the inhibitory signal programmed cell death protein 1 (PD-1) and the activation marker CD44. Moreover, we assessed CD154 after in vitro stimulation. PwMS had significantly increased frequencies of HLADR expressing B cells (97.6% vs. 93.3%, *p* = 0.020) and cT cells (7.8% vs. 3.7%, *p* = 0.004) compared with HCs (Figure 1e and Figure A1e). CD44 was expressed in over 90% of all the investigated lymphocytes without differences across the two cohorts (Figure 1f and Figure A1f). The proportions of CD28+ cT cells were significantly increased among pwMS (59.5% vs. 32.5%, *p* = 0.000128), while no differences were found for Th and B cells (Figure 1g and Figure A1g). Importantly, pwMS had significantly higher frequencies of Th cells expressing CD154+ compared with HCs (41.7% vs. 35.6%; *p* = 0.009; Figure 1h and Figure A1h). Finally, we found that significantly fewer B cells from pwMS contained the regulatory signal PD-1 (0.99% vs. 5.5%, *p* = 0.016; Figure 1i and Figure A1i), while the expression of PD-1 on T cells was not dysregulated in our MS cohort. In addition to differences in the frequencies, we found a significantly more intense signal for HLADR on the surface of B cells in pwMS (*p* = 0.001276; Figure 2e). The surface density of the other investigated CAMs and CoSs did not significantly differ between the two cohorts (Figure 2).

### 3.2. Cladribine-Associated Changes in Costimulatory and Adhesion Molecules

In the second part of this study, we evaluated if treatment with CLAD was associated with changes regarding the expression patterns of CAMs and CoSs on the surface of CD4+, CD8+ and CD19+ lymphocytes.

#### 3.2.1. B Cells

Treatment with CLAD was associated with significant changes in the surface profile of several CAMs and CoSs in B cells. Importantly, significant differences were observed only at 24 months after CLAD initiation.

**Figure 3:**
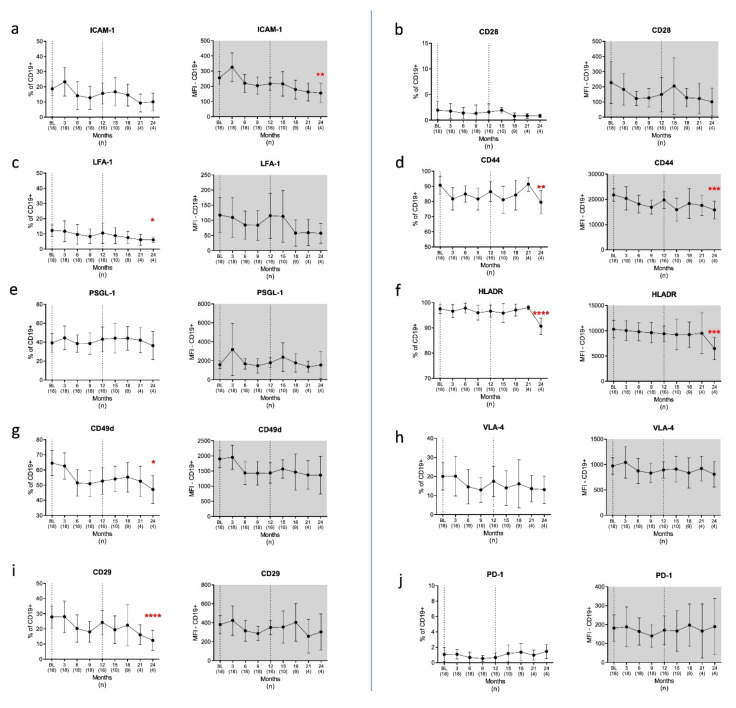
Adhesion and costimulatory surface proteins on B cells/CD19+ (**a**–**j**). Proportions of B cells expressing CAMs and CoSs (white graphs) as well as the intensity of expression (MFI) of positive cells (grey graphs) were evaluated by flow cytometry over a period of 24 months. We found decreased surface densities for ICAM-1 (**a**), CD44 (**d**) and HLADR (**f**) on B cells and a reduced frequency of B cells expressing LFA-1 (**c**), CD44 (**d**), HLADR (**f**), CD49d (**g**), and CD29 (**i**) at 24 months after CLAD initiation. Legend: BL = baseline; *n* = number of patients included at each time point. Data are shown as mean values ±SD. * *p* ≤ 0.05, ** *p* ≤ 0.01, *** *p* ≤ 0.001 and **** *p* ≤ 0.0001.

Regarding CAMs, the density of ICAM-1 was significantly reduced at 24 months (*p* = 0.004) and the frequency of ICAM-1 expressing B cells declined from 18.2 to 10.1% (*p* = 0.083, Figure 3a), clearly approaching values from HCs (8.8%). LFA-1 expressing B cells were reduced from 12.2% to 6.1% (*p* = 0.015, Figure 3c), reaching the levels of HCs (7.3%). Moreover, significantly lower proportions of B cells expressed CD49d and CD29 (*p* = 0.047 and *p* = 0.00007 respectively; Figure 3g,i), at the end of Year 2. The expression patterns of PSGL-1 and VLA-4 remained unchanged (Figure 3e,h). We next investigated the expression changes in CoSs during the observation period. No changes in the expression profile of CD28 were found (Figure 3b). Significant reductions were found for CD44 (Figure 3d) and HLADR (Figure 3f) expression in Month 24. Both proteins were reduced in frequency (*p* = 0.009 and *p* = 0.000003, respectively) as well as in terms of MFI (*p* = 0.0001 and *p* = 0.001, respectively). The proportions of HLADR+ B cells, which were found to be increased at BL compared with HCs, decreased from 97.6% (BL) to 90.7% (in Month 24), reaching HCs’ levels (93.3%) after 24 months. There was no impact on PD-1 (Figure 3j).

#### 3.2.2. T Cells

**Figure 4:**
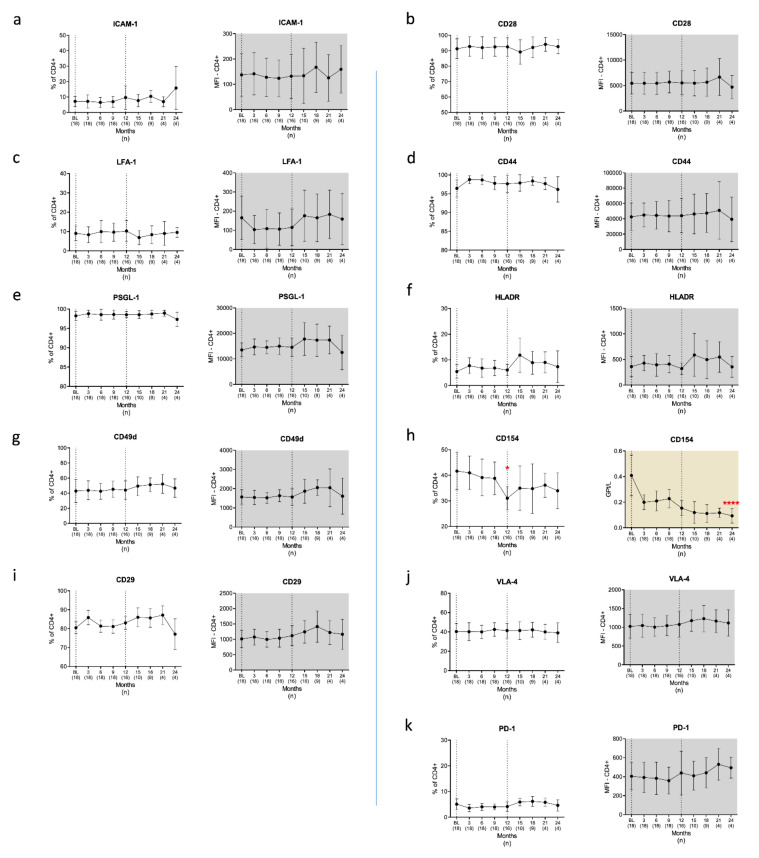
Adhesion and costimulatory surface proteins on the surface of T helper cells/CD4+ T lymphocytes (**a**–**k**). Blood was drawn before cladribine initiation (BL) and every 3 months over a period of 2 years and analysed by flow cytometry. CD154 expression decreased in relative as well as in absolute numbers (**h**). Legend: BL = baseline; *n* = number of patients included at each time point. Data are shown as mean values ±SD. * *p* ≤ 0.05 and **** *p* ≤ 0.0001.

**Figure 5:**
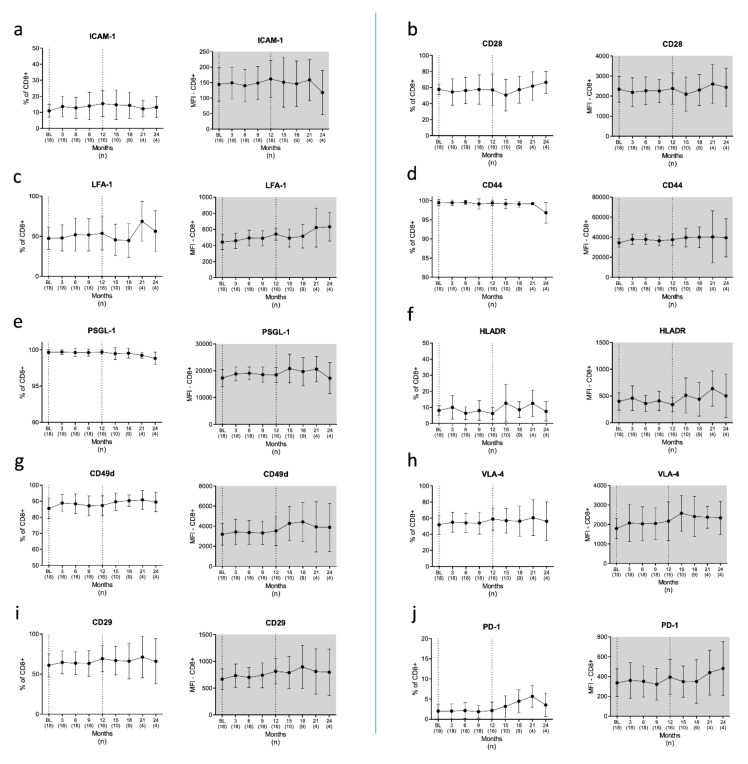
Adhesion and costimulatory surface proteins on the surface of cytotoxic T cells/CD8+ T lymphocytes (**a**–**j**). Blood was drawn before cladribine initiation (BL) and then quarterly over a period of 24 months. We found no CLAD-associated changes in the expression patterns of CD8+ cells as evaluated by flow cytometry. Legend: BL = baseline; *n* = number of patients included at each time point.

We next investigated the surface expression profiles of CAMs and CoSs on Th (Figure 4) and cT (Figure 5) lymphocytes. We found a significant decline in CD154 expressing Th cells in Month 12 (*p* = 0.02), but not at any of the other time points. At BL, 41.7% of the Th cells from pwMS expressed CD154. This immune cell subset was reduced to 31% in Month 12 after CLAD initiation (Figure 4h), recovering to 34% in Month 24; however, it was still below the frequency in HCs (35.6%). In absolute terms, the numbers of CD154+ CD4+ cells were reduced by 77% over the observational period (*p* = 0.000002). Apart from CD154 frequencies, we found no changes in the expression profiles of CAMs and CoSs in Th cells associated with CLAD treatment. Moreover, no CLAD-associated effects were observed regarding the surface expression of inflammatory and regulatory signals on cT cells during the 24 months of the investigation (Figure 5).

## 4. Discussion

In this study, we characterized MS-related differences in the pattern of proteins involved in adhesion and costimulation on the surface of immune cells and examined CLAD-associated changes in these over time. We report three main findings. First, we corroborate the selective pro-migratory and -inflammatory profile of CAMs and CoSs in pwMS. This signature is characterized by a higher percentage of ICAM-1 (CD19+, CD4+, CD8+), LFA-1 (CD19+, CD8+), HLADR (CD19+, CD8+), CD28 (CD8+) and CD154 (CD4+) expressing immune cells. Moreover, we observed an increased surface intensity staining for LFA-1 (CD8+) and HLADR (CD19+) in our MS cohort. Secondly, with regard to the markers studied, B cells were the main immune cell subset affected by CLAD therapy. We found decreased surface densities for ICAM-1, CD44 and HLADR on B cells and a reduced frequency of B cells expressing LFA-1, CD49d, CD29, CD44 and HLADR at 24 months after CLAD initiation. The third observation is that CLAD did not change the expression patterns of CAMs and CoSs in cT cells.

Our data emphasize the immune-driven pathogenesis of MS, as peripheral blood immune cells from pwMS carried a pro-migratory and activated surface profile. We observed HLADR overexpression in B cells, suggesting an abnormal antigen-presenting capacity in MS. Moreover, T and B cells from our MS cohort expressed higher amounts of ICAM-1 (CD4+, CD8+, CD19+) and LFA-1 (CD19+, CD8+). ICAM-1 on antigen-presenting B cells engages LFA-1 on Th cells. This cell–cell contact is essential for the Th–B cell crosstalk and for the ensuing antigen recognition [39]. Both molecules are additionally involved in lymphocyte migration into inflamed tissue [40]. Therefore, increased proportions of ICAM-1 and LFA-1 carrying immune cells may not only reflect an increased potential to infiltrate the CNS but also pathogenic interactions for the lymphocyte crosstalk. In addition to the aforementioned activated and pro-migratory immune cell surface expression profiles, we found reduced VLA-4 expressing Th cells in pwMS. As our pwMS had active disease at BL, the pool of peripheral VLA-4 expressing lymphocytes might have been diminished by attachment to their cognate receptors in brain endothelial or lymphoid tissue.

With regard to the potential immune reconstitution phenomena induced by CLAD, we found significant changes in CAMs and CoSs in the PBMC. Indeed, CLAD treatment was associated with a decreased density of ICAM-1, CD44 and HLADR on the surface of B cells. In addition, we observed significantly lower proportions of B cells with LFA-1, CD49d, CD29 and CD44 as well as HLADR surface expression. Importantly, all changes in B cells were detected at 24 months after CLAD initiation but not at earlier time points, indicating that the observed CLAD-associated surface modifications represent long-term consequences. Our study provides evidence that MS-specific dysregulation of ICAM-1, LFA-1 and HLADR expression in B cells and the increased frequencies of CD154 expressing Th cells are corrected by treatment with CLAD. This observation may be related to the consequences of immune reconstitution and need to be seen as indirect effects, as they do not reflect the depletion and reconstitution kinetics reported for CLAD. We have shown earlier that CLAD reduces the number of memory B cells (CD19+CD27+) and Th17 cells (CD4+IL17+) within 6 months after administration, followed by a stepwise repopulation towards the end of each treatment year [30].

The involvement of CAMs and CoSs in the immunopathogenesis of MS was supported by previous reports on the effects of various DMTs on their expression, and can now be extended for CLAD. PwMS exhibit elevated frequencies of CD154 expressing Th cells [41], which were suppressed by interferon-beta [42]. Fingolimod downregulated ICAM-1 expression and increased the integrity of the BBB in an animal model [43]. In line with our analysis, DMF decreased HLADR expression in B cells [28]. Natalizumab not only blocks VLA-4 but also induces downregulation of CD49d [44].

We observed reductions in CD44 expression by B cells at 24 months following CLAD therapy. CD44 is an activation marker, which exhibits stimulatory as well as migratory capacities [45]. CD44 is chronically overexpressed by glial cells in demyelinated MS lesions [46]. Moreover, CD44 knockout mice experience milder EAE, implicating a direct contribution to autoimmune processes. The effect on EAE was explained by a shift from inflammatory Th1 and Th17 cells towards anti-inflammatory Th2 and regulatory T cells [47]. CD44 is expressed by APCs within the IS and contributes to T cell activation and interferon gamma (IFN-γ) production [48]. Of note, IFN-y is produced by Th1 and Th17.1 lymphocytes, and evidence for a pathogenic role in MS is especially strong for the latter [49,50]. Decreased expression of CD44 by B cells within the IS could therefore limit the proliferation of autoreactive T cells.

We did not observe changes in the studied surface molecules in cT following CLAD therapy. CT cells play a vital role in virus clearance. Together with the modest CLAD effect on cT in terms of depletion [30,33], our findings indicated no consequences on their structural properties with regard to the expression of CAMs and CoSs and any subsequent limitations to combat infection [51,52].

While the depleting effect on B cells has been well described in the literature and peaks early after CLAD administration (nadir at 13 weeks [33]), significant alterations in CAMs and CoSs occur later, at 24 months after treatment initiation. From our in vivo protocol, we cannot draw conclusions on the underlying mechanism impacting on the surface expression patterns on B cells. One possible explanation could be that the observed reductions in CAMs and CoSs resulted from reduced costimulation following B and T cell depletion. Even though our data suggest that CLAD treatment primarily impacts on the surface expression profile of B cells, we cannot exclude alterations in specific lymphocyte subsets. It would therefore be of great interest to investigate CAMs and CoSs in further lymphocyte phenotypes (e.g., memory B cell subsets, central memory T cells, Th17 cells and regulatory subsets) and study a larger cohort and stratify subjects for clinically stable vs. active disease.

We observed a mid-term effect on CD154 expressing Th cells. CD154 (CD40L) binds CD40 on B cells within the IS. This ligation induces T-cell-mediated B cell stimulation and maturation [53]. Interestingly, it has been shown that this interaction is altered in MS, and that B cells from pwMS proliferate when stimulated with CD154 [6]. The CD154/CD40 pathway also plays a crucial role in the development of EAE [54,55], and disrupting this communication by blocking antibodies had beneficial effects not only in EAE [54,56,57] but also in other autoimmune disease models [58,59]. Therefore, reduced frequencies of CD154 expressing Th cells may abrogate the autoreactive potential of the IS. Future studies are warranted to further address CD154/CD40 dysregulation in MS and to determine whether CLAD selectively targets CD154 expression in different T helper cell phenotypes.

Our findings have to be interpreted with care, as the study is based on a small sample size. Moreover, all patients had an active disease at the time of recruitment, and therefore a reduction in CAMs and CoSs over time could represent the natural course associated with a stable clinical course induced by CLAD. We cannot exclude that the process of freezing and thawing impacted on the expression of the adhesion molecules. In order to reduce the data acquisition bias, all samples were processed in a single batch and did not undergo repeated freeze–thaw cycles.

## 5. Conclusions

In conclusion, immune reconstitution by oral CLAD is associated with modification of the pro-migratory and -inflammatory surface patterns of CAMs and CoSs in immune cell subsets in pwMS. This observation pertains primarily to B cells, which are key cells underlying MS pathogenesis.

## Figures and Tables

**Table 1:** Demographics and baseline lymphocyte subsets of the study populations.

	pwMS *n* = 18	HCs *n* = 10
Sex, F/M	15/3	7/3
Age, mean years ± SD (range)	37.4 ± 11.7 (20–57)	37.0 ± 12.9 (25–60)
EDSS BL, mean ± SD	2.22 ± 1.7	-
EDSS EOS, mean ± SD	2.08 ± 2.0	-
MS duration, mean years (range)	8.8 (0–25)	-
Lymphocyte subsets at BL:		
% of CD4+ cells among lymphocytes (±SD)	44.2 (13.7)	41.1 (11.2)
% of CD8+ cells among lymphocytes (±SD)	24.3 (9.2)	23.8 (7.2)
% of C19+ cells among lymphocytes (±SD)	12.1 (6.8)	14.3 (5.9)

Group comparisons were performed by Student’s *t*-test; no significant differences were detected for age and sex. MS: multiple sclerosis; pwMS: patients with multiple sclerosis; HCs: healthy controls; F: female, M: male; SD: standard deviation; BL: baseline; EOS: end of study.

## Data Availability

The data that support the findings of this study are available on reasonable request from the corresponding author.

## Appendix A

**Table A1:** Site/year of patient recruitment, clinical characteristics and pre-treatment.

Patient	Site	Year	Diagnosis	MS Duration (Months)	Pre-Treatment *	Relapses **	EDSS BL	EDSS EOS
#1	Austria	2017	RRMS	6	glatiramer acetate	1 (Month 13)	1.0	1.0
#2	Austria	2018	RRMS	18	-	-	2.5	2.5
#3	Austria	2018	RRMS	44	daclizumab	-	2.0	2.5
#4	Austria	2018	RRMS	95	interferon-beta	-	3.5	3.0
#5	Austria	2018	RRMS	47	dimethyl fumarate	-	1.5	0
#6	Austria	2018	RRMS	301	fingolimod	2 (Months 3 and 6)	3.5	4.0
#7	Austria	2018	RRMS	98	glatiramer acetate	-	1.5	1.0
#8	Austria	2018	RRMS	184	fingolimod	-	1.0	1.5
#9	Austria	2018	RRMS	33	dimethyl fumarate	1 (Month 13)	2.0	2.0
#10	Austria	2018	RRMS	18	interferon-beta	-	2.0	0
#11	Austria	2019	RRMS	24	-	-	0	0
#12	Austria	2019	RRMS	113	glatiramer acetate	-	0	0
#13	Austria	2019	RRMS	107	teriflunomide	-	0	0
#14	Austria	2019	RRMS	207	IVIG	-	3.0	2.5
#15	Germany	2018	SPMS	252	daclizumab	-	4.5	5.5
#16	Germany	2018	RRMS	96	fingolimod	-	3.0	3.5
#17	Germany	2018	SPMS	143	-	-	7.0	7.0
#18	Germany	2018	RRMS	110	-	-	2.0	1.5

CLAD: cladribine; RRMS: relapsing remitting multiple sclerosis; SPMS: secondary progressive multiple sclerosis; BL: baseline; EOS: end of study. * MS drugs administered within 12 months after CLAD initiation. ** Number of relapses during the study period and occurrence (months after CLAD initiation).
